# Methodological Considerations on the Prognostic Value of HALP Score in Rheumatic Mitral Stenosis

**DOI:** 10.1002/clc.70327

**Published:** 2026-04-23

**Authors:** Oğuzhan Yılmaz, Serkan Ünlü, Özden Seçkin

**Affiliations:** ^1^ Department of Cardiology Gazi University Faculty of Medicine Ankara Türkiye

Dear Editor,

We read with great interest the recent article by Kaya et al. evaluating the prognostic value of the hemoglobin–albumin–lymphocyte–platelet (HALP) score in patients with rheumatic mitral stenosis (RMS) [1]. The authors should be commended for addressing a clinically relevant question in a population in which simple and accessible risk markers may be of practical value. However, we believe that the clinical interpretability of the findings is limited by the construction of the composite endpoint, the modest discriminatory performance of HALP, and internal inconsistencies in the reporting of baseline characteristics.

First, the definition of the primary endpoint deserves careful consideration. Major adverse cardiac and cerebrovascular events (MACCE) were defined as death, myocardial infarction, stroke, and mitral valve interventions, including percutaneous mitral balloon valvuloplasty and surgery [1]. In clinically significant RMS, however, the decision to proceed with intervention is not determined solely by spontaneous disease progression; it may also be influenced by symptom burden, valve anatomy, hemodynamic severity, embolic risk, and local expertise [2]. Therefore, combining intervention‐driven outcomes with hard clinical endpoints may complicate the interpretation of the observed association between HALP score and prognosis.

Second, the reported prognostic performance of HALP appears limited. Receiver operating characteristic analysis yielded an area under the curve of 0.644, with a sensitivity of 60.4% and a specificity of 38.1%. In addition, Cox regression analysis did not demonstrate an independent association between HALP score and outcomes (OR 0.976, 95% CI 0.950–1.003; *p* = 0.076) [1]. Hematological and nutritional indices have been associated with outcomes in rheumatic mitral valve disease [3]. Similarly, the HALP score has been linked to mortality in patients with coronary heart disease [4]. In a broader valvular heart disease population, HALP has also shown prognostic value in a large‐scale cohort [5]. Taken together, these data suggest that in RMS, HALP may be more appropriately interpreted as a complementary marker rather than a standalone prognostic tool. In this regard, more detailed reporting of the multivariable model would improve interpretability, particularly whether established determinants such as atrial fibrillation, mitral stenosis severity, left atrial enlargement, pulmonary artery pressure, and concomitant valvular involvement were included in risk adjustment.

Third, some internal inconsistencies in the manuscript appear to merit clarification. Chronic kidney disease is listed among the exclusion criteria in the Methods section, yet Table 1 reports three patients with chronic kidney disease. In addition, the reported rhythm categories do not sum to the total study population, and several percentages in Table 1 appear inconsistent with the stated cohort size. Such discrepancies may affect the interpretation of the baseline characteristics and should be clarified.

In summary, this study adds to the growing literature on inflammation‐ and nutrition‐based markers in valvular heart disease. Nevertheless, separate analyses for hard clinical outcomes and intervention‐related outcomes, together with clarification of baseline data consistency and multivariable adjustment, would strengthen the clinical interpretation of the findings. Until these issues are clarified, HALP should be interpreted as a complementary marker rather than a standalone prognostic tool in patients with RMS.

## Conflicts of Interest

The authors declare no conflicts of interest.

## Data Availability

Data sharing is not applicable to this article as no data sets were generated or analyzed during the current study.
